# Dysbiosis of the Subgingival Microbiome and Relation to Periodontal Disease in Association with Obesity and Overweight

**DOI:** 10.3390/nu15040826

**Published:** 2023-02-06

**Authors:** Betul Rahman, Farah Al-Marzooq, Hiba Saad, Dalenda Benzina, Sausan Al Kawas

**Affiliations:** 1 Department of Preventive and Restorative Dentistry, College of Dental Medicine, University of Sharjah, Sharjah 27272, United Arab Emirates; 2 Department of Medical Microbiology and Immunology, College of Medicine and Health Sciences, UAE University, Al Ain 15551, United Arab Emirates; 3 Department of Oral and Craniofacial Health Sciences, College of Dental Medicine, University of Sharjah, Sharjah 27272, United Arab Emirates

**Keywords:** subgingival microbiome, oral dysbiosis, obesity, overweight, periodontitis, 16S rRNA sequencing

## Abstract

Obesity causes gut dysbiosis; nevertheless, little is known about the oral microbiome. We aimed to identify differences in the subgingival microbiota influenced by body weight and periodontal status. Patients (*n* = 75) recruited at the University Dental Hospital Sharjah, United Arab Emirates, were distributed into three equal groups (healthy weight, overweight, and obese) sub-divided into having either no-mild (NM) or moderate-severe (MS) periodontitis. Subgingival plaques were collected. Microbiota were identified by 16S rRNA sequencing using nanopore technology. Linear discriminant analysis demonstrated significant bacterial biomarkers for body weight and periodontal health. Unique microbiota signatures were identified, with enrichment of periopathogens in patients with MS periodontitis (*Aggregatibacter actinomycetemcomitans* in obese, *Tannerella forsythia* and *Treponema denticola* in overweight, *Porphyromonas gingivalis* and *Fusobacterium nucleatum* in healthy weight), thus reflecting differences in the microbiota affected by body weight. Other pathogenic bacteria, such as *Salmonella enterica* and *Klebsiella pneumoniae*, were enriched in overweight subjects with NM periodontitis, suggesting an increase in the relative abundance of pathogens even in patients with good periodontal health if they were overweight. Alpha and beta diversities were significantly different among the groups. Dysbiosis of the subgingival microbiota in obese and overweight individuals was associated with increased prevalence and severity of periodontal disease, which was correlated with the body mass index. This study highlights the immense importance of the oral microbiome and the need for lifestyle and dental interventions to resolve oral dysbiosis and restore normal homeostasis.

## 1. Introduction

Obesity is a global public health issue. In the Middle East, the prevalence of obesity and overweight was estimated to be 21.17% and 33.14%, respectively, according to a recent systematic review and meta-analysis of 101 studies with 698,905 participants [1]. Obesity can cause various health diseases in different organs and systems in the body, such as type 2 diabetes, cardiovascular diseases, and some types of cancer [2]. It is also recognized as a cause of diseases in the oral cavity [3]. The oral cavity hosts one of the most diverse microbial communities within the human body. Nowadays, the oral microbiome is considered to be a key determinant of oral and systemic health [4]. The microbiota can influence the host’s metabolic functions directly by affecting energy and nutrient availability or indirectly via modulation of signaling pathways through bacterial by-products as short-chain fatty acids [5]. Thus, it is important to analyze microbiota alteration in obese and overweight populations to understand the changes in the microbiome and its relationship with various diseases, including those affecting the oral cavity.

Periodontitis is a polymicrobial immunoinflammatory disease that can result in tooth loss. According to the latest classification of periodontal diseases based on the consensus of the 2017 World Workshop on the Classification of Periodontal and Peri-Implant Diseases and Conditions, periodontitis is described by using staging and grading of the disease. The stage is basically determined by the severity of disease at the initial examination besides the expected complexity of disease management. It incorporates the extent and distribution of the disease in the dentition. The grade stipulates additional information about the biological features of the disease based on a historical evaluation of the periodontitis progression rate, considering the risk for future progression and appraisal of the risk that periodontitis or its treatment may adversely affect the general health of the patient [6]. It focuses on the role of periodontitis as the second most frequent factor (following obesity), which is well known as a changeable contributor to systemic inflammatory load. Obesity, nutrition, certain genetic factors, and physical activity are emerging risk factors that may contribute to the risk of developing periodontitis in the future [7].

Oral pathobionts coupled with ongoing gingival inflammation are critical for the initiation and progression of periodontal disease [4]. Red complex bacteria (*Porphyromonas gingivalis, Treponema denticola*, and *Tannerella forsythia*) represent the most important pathogens in adult periodontal disease. Additionally, the abundances of *Fusobacterium nucleatum*, *Prevotella species*, *Eikenella corrodens*, *Peptostreptococcus micros*, and *Campylobacter rectus* are increased in deep periodontal pockets and implicated as possible periodontal pathogens [8]. High bacterial counts of multiple organisms were found in deeper pockets, suggesting that some bacteria may cause destruction of the periodontal tissue in a cooperative manner [9].

Abnormal changes in microbiome composition and community structures are linked to the dysbiosis observed in periodontal disease. Several studies have shown an imbalanced immune-inflammatory response in obese individuals [10,11,12]. Persistent and imbalanced inflammation caused by obesity may result in an altered periodontal microbiota, which effects the composition of the subgingival biofilm [13]. Thus, it is crucial to investigate the microbiota of subjects with obesity and periodontal disease to understand the link between these conditions.

Goodson et al. (2009) explored the association between obesity and 40 different oral microorganisms, reporting that a subset of microorganisms were associated with weight gain [14]. A randomized controlled trial showed that intensive periodontal treatment reduced systemic inflammation and improved the lipid profile [10]. On the other hand, a recent population-based study conducted in Australian adults failed to show a significant association between obesity and periodontitis based on pocket depth and clinical attachment loss [15]. Other studies found a debatable association between periodontal disease and several metabolic risk factors. However, the studies assessed periodontal disease using known clinical methods (clinical attachment loss, periodontal pocket depth, and radiographic bone loss) without looking into the periodontal microbial composition [10,16,17,18,19,20]. In the case of non-advanced stages of periodontal disease, these clinical assessment measures become more complex to determine, especially since conditions such as gingival bleeding can be reversed by good oral hygiene [17]. Therefore, the likelihood of underestimating the association between the periodontal bacterial profile and obesity cannot be ruled out [10]. Hence, the periodontal bacterial profile may be a good diagnostic marker at early stages of the disease [18,21]. Moreover, the relationship between periodontal disease and obesity will become substantially stronger when clinical periodontal disease ascertainment is coupled with profiling of periodontal microorganisms.

The overall objective of this study was to investigate the possible influence of obesity on periodontal disease by assessing subgingival microbiome community composition and taxon abundance by bacterial 16S rRNA gene sequencing of subgingival plaque. The specific objectives of this study were: to investigate the relationship between severity of periodontitis and anthropometric measures of adiposity; to identify clusters of subgingival periodontal microorganisms found in obese, overweight, and normal weight periodontitis patients; and to investigate the possible shift in the subgingival microbiota of obese subjects who do have no periodontitis towards periodontopathogens that might increase the risk of periodontitis.

## 2. Materials and Methods

### 2.1. Ethical Approvals

This study was approved by the Research Ethics Committee of the University of Sharjah, UAE (REC-16-10-30-03) and conducted in accordance with the Declaration of Helsinki. Adult patients seeking dental treatment in the University Dental Hospital Sharjah (UDHS) who agreed and gave written consent to participate in the study were recruited.

### 2.2. Study Design

This was a cross-sectional study conducted over a period of 2 years. The sample size calculation was performed using the online tool (https://clincalc.com/stats/samplesize.aspx; access date: 9 Feburary 2020). For achieving 90% power; β = 0.1, and α = 0.05 at a 2-tailed significance level of 95%, the sample size for each independent study group was 29 patients. However, we were able to recruit 25 patients per group in the three groups (obese, overweight, and healthy weight) for a total of 75 patients included in this study.

Blinding of the outcome assessors was performed to reduce the potential bias of investigators [22]. Dentists examining the patients were not aware of the study goals while recruiting patients and collecting subgingival plaque samples. The same approach was followed for laboratory testing; the samples were provided to the lab with codes without revealing the oral health of patients or information related to body weight. Finally, results were received and lab data were correlated with clinical data by the project main investigators.

### 2.3. Inclusion Criteria

Patients aged between 18–60 years and having at least 10 teeth in their mouths were considered. Edentulous patients, patients who received any periodontal treatment during the last 3 months, and patients who were on antibiotic therapy during or within 3 months of the study were excluded because any type of periodontal treatment including subgingival instrumentation and/or antibiotic treatment could modify the composition of the subgingival microflora. Female patients who were pregnant were excluded due to hormonal changes and weight gain in pregnancy. Medically compromised patients were excluded, including patients with uncontrolled diabetes. Patients with ongoing orthodontic treatment were also excluded since fixed orthodontic appliances make oral hygiene practice difficult for the patient, thus patients have more gingival inflammation than normal during this treatment period.

### 2.4. Anthropometric Measurements

Weight was measured using a digital weighing scale and height with a meter. The body mass index (BMI) was computed as weight in kilograms divided by height in meters squared (kg/m^2^). Finally, the cases were categorized into three groups (*n* = 25 each) including normal weight (BMI: 18.5–24.9), overweight (BMI: 25–29.9), and obese (BMI ≥ 30) [23]. BMI was assessed as an indicator of overall adiposity. The waist and hip circumferences were also assessed using a measuring tape and the waist-to-hip ratio (WHR) was calculated as the waist circumference divided by the hip circumference.

### 2.5. Oral Examination and Periodontal Assessment

We performed a comprehensive periodontal examination of the 75 participants. The oral examination was performed using a mouth mirror, William’s periodontal probe, and a tweezer. All examinations were performed by one experienced dentist who also enrolled the patients and filled out the case sheets. Presence of dental plaque on tooth surfaces was recorded when clearly visible and expressed using the visible plaque index (VPI) [24].

Gingival inflammation was assessed based on bleeding on probing (BOP) of the gingival sulcus of all teeth (wisdom teeth excluded) at six sites of each tooth. The proportion of surfaces (%) with visible dental plaque and gingival inflammation was calculated for each subject [25].

The Probing Pocket Depth (PPD) and Clinical Attachment Loss (CAL) at six sites on all teeth were assessed. The severity of periodontitis was diagnosed for each patient based on the latest periodontal disease definition and classification established during the 2017 world workshop. The patient was diagnosed as having periodontitis if interdental CAL was measurable at two or more non-adjacent teeth or CAL was 3 mm or more buccally or lingually with more than 3 mm deep pocket in the same location at two or more teeth [7].

When interdental CAL at the site of greatest loss was 1–2 mm, the patient was diagnosed as having Stage I periodontitis (mild or initial periodontitis) with no tooth loss due to periodontitis and the greatest probing depth was 4 mm or less. When interproximal CAL at the site of maximum loss was 3–4 mm, no tooth loss because of periodontitis, and the maximum probing pocket depth was 5 mm or less, the patient was diagnosed as having Stage II periodontitis (moderate periodontitis). When interdental CAL at the site of maximum loss was 5 mm or more, with loss of 4 teeth or less as a result of periodontitis, and a probing depth of 6 mm or more, the patient was diagnosed as having Stage III periodontitis (severe periodontitis). When a patient had lost 5 or more teeth due to periodontitis as well as having Stage III criteria and needed complex oral rehabilitation, he/she was diagnosed as having Stage IV periodontitis (advanced periodontitis) [7]. Based on the criteria above, we sorted the participants into two groups, including subjects with no or mild periodontitis (NM) at Stage I or below and subjects with moderate to severe/advanced periodontitis (MS) at Stages II to III/IV.

### 2.6. Subgingival Plaque Samples

The subgingival plaque samples were collected at the same time of day (in the afternoon, approximately 5–7 h after tooth brushing). Samples were collected from each subject by inserting a total of 16 sterile endodontic paper points (size 30; two paper points per site; 8 sites) into the gingival sulci or periodontal pocket for 10 s, following isolation and supragingival plaque removal [26]. The subgingival samples from periodontally healthy subjects were collected by passing the paper points across each gingival sulci and pooled from eight teeth of quadrants 1 and 3 (incisor, canine, premolar, and molar). In periodontitis patients, subgingival samples were collected and pooled from the deepest PPD sites in each quadrant (a total of eight non-adjacent proximal sites). Samples were placed in 1.5 mL micro-centrifuge tubes with 300 μL of phosphate buffer and put on dry ice, then transferred to −80 °C freezer until further analysis [26].

### 2.7. Nanopore Sequencing of 16S rRNA Gene from the Subgingival Plaques

DNA extraction was performed using the Epicentre MasterPure™ DNA Purification Kit (Epicenter, Middleton, WI, USA), according to the manufacturer’s recommendations. The quality and quantity of the extracted DNA was checked using a NanoDrop (Colibri Microvolume Spectrometer; Titertek-Berthold, Germany). Amplification of the entire (~1500 bp) 16S rRNA gene was performed using the 16S Barcoding Kit (SQK-RAB204; Oxford Nanopore Technologies, Oxford, UK) and LongAmp™ Taq 2 × Master Mix (New England Biolabs, UK) with 1 µg of input DNA per sample. Purification of the PCR products was performed using AMPure XP (Beckman Coulter, CA, USA) followed by quantification using a Qubit 4 fluorometer (Thermo Fisher Scientific, Waltham, MA, USA). Equimolar amounts of the amplification products were pooled together, then a total of 100 ng DNA of the pooled sample was used for library preparation. The microbiota was analyzed using third-generation sequencing with Nanopore technology on a MinION device (Oxford Nanopore Technologies, UK). MinION™ sequencing was performed using R9.4 flow cells (Oxford Nanopore Technologies, UK) according to the manufacturer’s instructions. MinKNOW version 2.0 (Oxford Nanopore Technologies, UK) was used for live base calling and data acquisition. The raw data were converted into FASTQ format using Guppy v3.4.4, followed by demultiplexing and removal of nanopore and adaptor sequences. The FASTQ files were analyzed on the Nanopore EPI2ME platform with a default minimum Q score of 7. Preliminary bacterial identification was performed via the ‘What’s in my Pot?’ (WIMP) workflow provided by Oxford Nanopore Technologies (UK). Reads assigned to all targets were re-analyzed by the Kraken taxonomic sequence classification system using Partek^®^ Genomics Suite^®^ software (Copyright^©^ 2022; Partek Inc., St. Louis, MO, USA). The numbers of reads assigned per taxon were counted and the relative abundance of reads per taxon were used for separate downstream analysis, as described in our previous publications [27,28].

### 2.8. Statistical and Bioinformatics Analyses

SPSS Statistics 28 software (IBM SPSS^®^ Statistics, Armonk, NY, USA) was used for statistical analysis. Subjects’ characteristics were described using frequency distribution for categorical variables and mean and standard deviation for continuous variables. The chi-square test was used to determine whether there was a significant relationship between categorical variables.

Anthropometric measures, periodontal health parameters, microbiota relative abundance, and alpha diversity indices were compared using non-parametric tests, including Kruskal–Wallis (>2 groups comparison) or Mann–Whitney U (two groups comparison) tests for samples grouped based on obesity and periodontal health. In order to compare variables within sub-groups (obesity and periodontal health), SPSS files were split and Kruskal–Wallis (>2 groups comparison) or Mann–Whitney U (two groups comparison) tests were used. Correlations between relative abundance of taxa and DMFT were calculated using Spearman correlation coefficients. All statistical tests were two-tailed. A *p*-value < 0.05 was considered statistically significant.

The Microbiome Analyst 2.0 platform (McGill, Canada) [29] was used to determine linear discriminant analysis (LDA) effect size (LEfSe) in order to detect biomarkers from the microbial profiles [30]. Shannon and Simpson diversity indices, Chao1-type estimator for diversity from abundance data, ACE (Abundance-based Coverage Estimator), Pielou’s index of species evenness, and observed species for counts of unique OTUs in each sample were used to estimate microbiota α-diversity, richness, and evenness.

For β-diversity (between groups comparison), Bray–Curtis and Jaccard distance matrices were used to assess the dissimilarity of samples and visualized through principal coordinate analysis (PCoA) and dendrograms. Permutation-Based Analysis of Variance (PERMANOVA) was used to compare beta diversity indices among the groups [29].

Venn diagrams were generated to show the shared and unique operational taxonomic units (OTUs) among groups, based on the occurrence of OTUs in a group regardless of their relative abundance using the Venny bioinformatics tool (version 2.1). Heatmaps were constructed using R version 4.0.1 (package: gplots; function: heatmap.2).

## 3. Results

### 3.1. Demographic and Clinical Characteristics of Participants

In this study, a total of 75 subjects were included, including 16 females (21.3%) and 59 males (78.7%). Other demographic and clinical information are included in Table 1.

As shown in Table 1, 53.3% of the participants had no-mild periodontitis (NM), while 46.7% had moderate-severe periodontitis (MS). When these participants were compared, significant differences were found in their BMI and WHR in addition to their periodontal parameters, including PD and CAL (*p* < 0.01). Accordingly, participants with MS periodontitis had significantly higher values for the aforementioned variables (Table 2).

When the participants were compared based on their BMI groups, they were significantly different in terms of their PD but not CAL (*p* < 0.01). This reflected the presence of a significant association between obesity and poor periodontal health. This finding was supported by the results of the correlation analysis, whereby a significantly positive correlation (*p* < 0.01) was found between body weight, BMI, and PD as an indicator of periodontal health.

### 3.2. Bacterial Abundance and Distribution

**Figure 1:**
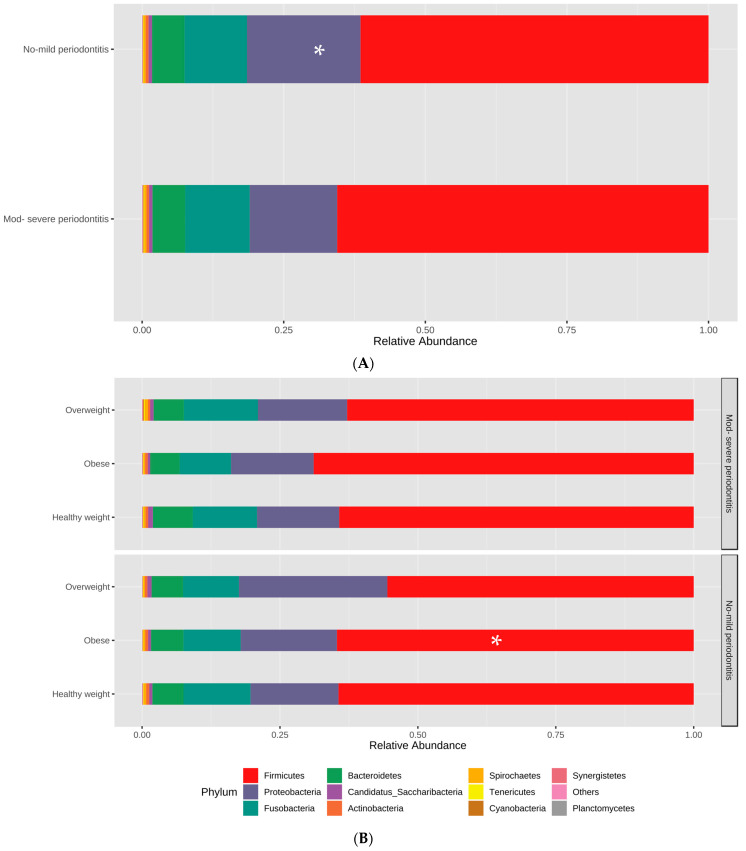
Relative abundance of phyla detected in the participants grouped based on the periodontal health status (**A**), then sub-grouped based on BMI (**B**).

The subgingival microbiome was investigated in all 75 subjects. The identified phyla are shown in Figure 1.

In total, an average of 30 phyla, 563 genera, and 1227 species were identified in the samples. In terms of total counts of these taxonomic groups, non-significant variations were noted when subjects were grouped based on body weight or periodontal health; however, the composition and relative abundance of microbiota were variable, as shown below.

*Firmicutes* was the most abundant phylum in this study, with a relative abundance of 64.6 ± 14.4%. Other major phyla detected were *Proteobacteria* (17.6 ± 11.3%), *Fusobacteria* (10.7 ± 7.5%), and *Bacteroidetes* (5.4 ± 3.64%), among others.

When the study groups were compared, a significant difference was found in one phylum, *Proteobacteria*, with significantly higher abundance (*p* < 0.05) in subjects with NM periodontitis, as shown in Figure 1.

When considering both BMI and periodontal health, the phylum *Firmicutes* was significantly higher in abundance (*p* < 0.05) in obese subjects than in overweight subjects, especially in subjects with NM periodontitis, while other phyla were not significantly different among the groups, as shown in Figure 1B. The *Firmicutes/Bacteroidetes* ratio was not significantly different among subjects grouped based on BMI or oral health (*p* > 0.05).

**Figure 2:**
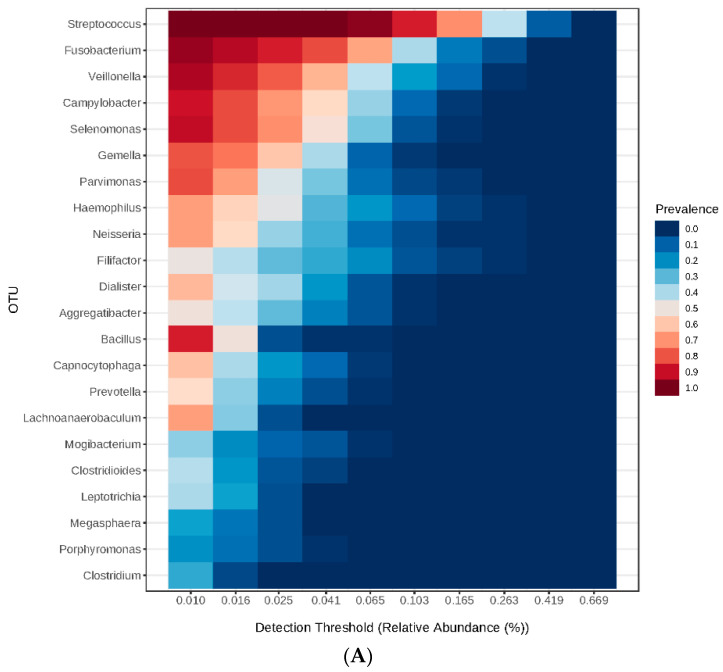

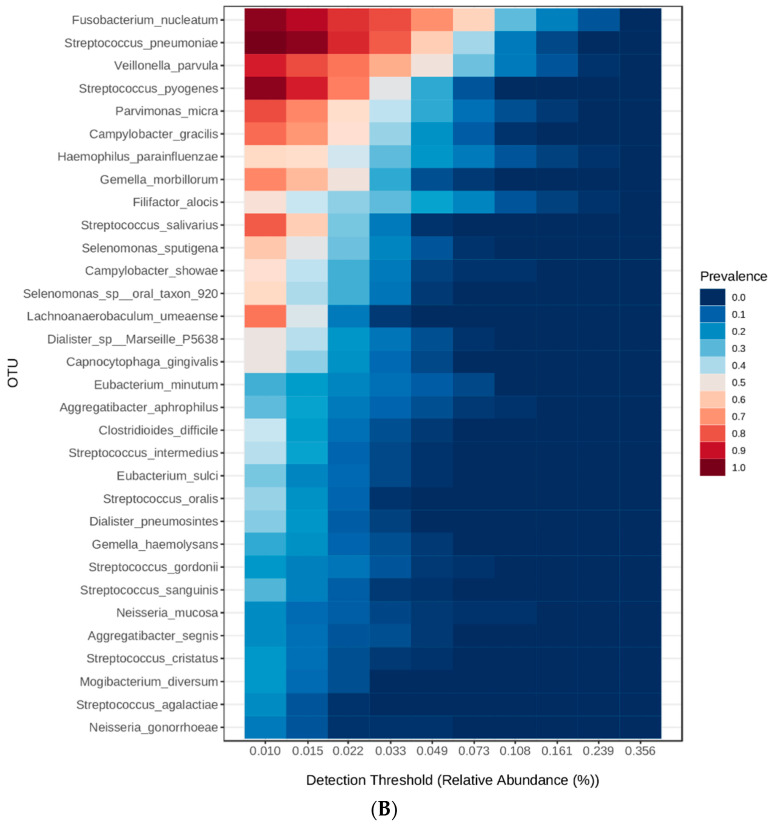
Heatmaps representing the core microbiome at genus level (**A**) and species level (**B**) detected among the study participants.

The core genera detected in the samples are summarized in Figure 2, which shows the top 22 genera and their detection threshold (relative abundance). As shown in Figure 2A, *Streptococcus* was the most prevalent genus (25.29 ± 13.54%), followed by *Fusobacterium* (10.46 ± 7.99%), *Veillonella* (7.4 ± 6.25%), *Campylobacter* (5.5 ± 4.18%), *Selenomonas* (5.05 ± 3.78%), and other genera detected with less than 5% abundance. As for species (Figure 2B), the top detected species were *Fusobacterium nucleatum* (9.78 ± 7.7%), *Streptococcus pneumoniae* (6.68 ± 4.14%), *Veillonella parvula* (6.16 ± 5.35%), and other species detected with abundance less than 5% abundance. Subsequently, the genera and species were analyzed and compared to identify significantly different taxa between the study groups.

### 3.3. Linear Discriminant Analysis (LDA) Effect Size (LEfSe)

To identify the featured taxa associated with obesity, periodontal health, and both factors, we performed linear discriminant analysis (LDA) on the microbial abundance profiles at the genus and species levels. These taxa can distinguish each specific study group from the other groups based on high abundance [31].

**Figure 3:**
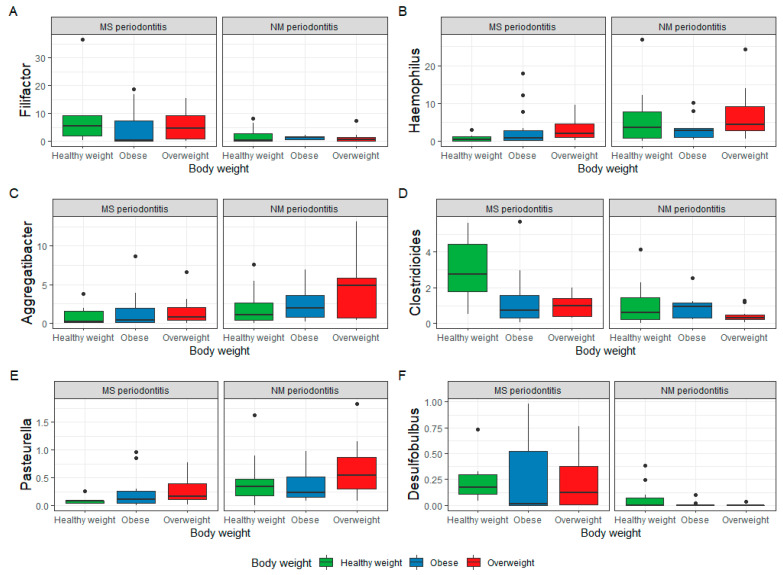
Top 6 genera with significant variations among the study groups (LDA score above 4) based on the linear discriminant analysis (LDA) effect size (LEfSe) for genera with significantly different abundance among groups differentiated on the basis of periodontal health status and BMI. Box plots show Q1-median-Q3 with range. Black dots are outlier values.

**Figure 4:**
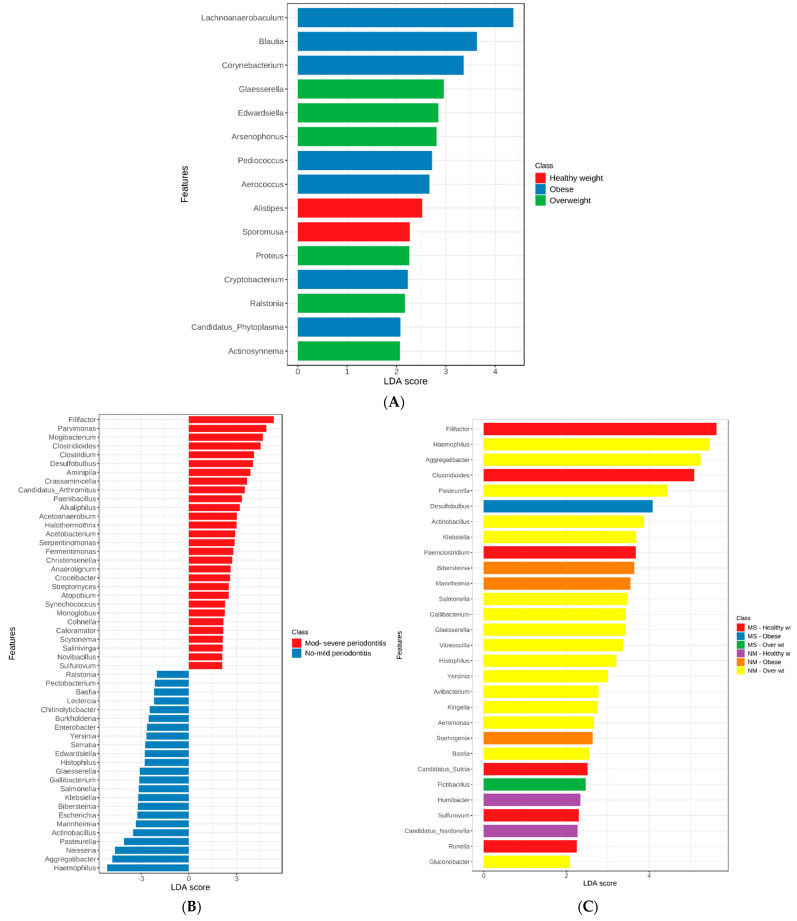
Genera detected by linear discriminant analysis (LDA) effect size (LEfSe) in subjects grouped based on their BMI (**A**), periodontal health status (**B**), and considering both factors (**C**). The genera shown are those that were significantly different among the groups ranked based on LDA score. Horizontal bars represent the effect size for each taxon.

When the genera were compared among the study groups, significant differences were detected for multiple genera (*p* < 0.05). Figure 3 shows the top 6 genera with significant variations among the study groups (LDA score > 4). *Filifactor* and *Clostridioides* were significantly more abundant in subjects with healthy weight and MS periodontitis, while *Aggregatibacter, Pasteurella*, and *Haemophilus* were significantly more abundant (*p* < 0.05) in overweight subjects with NM periodontitis. Multiple other genera, including pathogenic microorganisms such as *Klebsiella* and *Salmonella*, had lower LDA scores (>2). *Desulfobulbus* was the only significantly increased genus in obese subjects with MS periodontitis (*p* < 0.05). Figure 4 shows the LDA scores for significantly different genera in subjects grouped based on their BMI (A), periodontal health (B), and both factors (C).

It is obvious that periodontal health status had more impact on the genera in the subgingival microbiome, with more differentially abundant genera identified in subjects with NM than in those with MS periodontitis. As for genera in subjects grouped based on BMI, overweight and obese participants had more differentially abundant genera than subjects with healthy weight. When both periodontal health status and BMI were considered (Figure 4C), it is obvious that overweight subjects with NM periodontitis had the highest number of unique genera (*n* = 16) compared to the other groups, while 1–6 genera were unique in subjects with MS periodontitis grouped based on BMI (*p* < 0.05).

**Figure 5:**
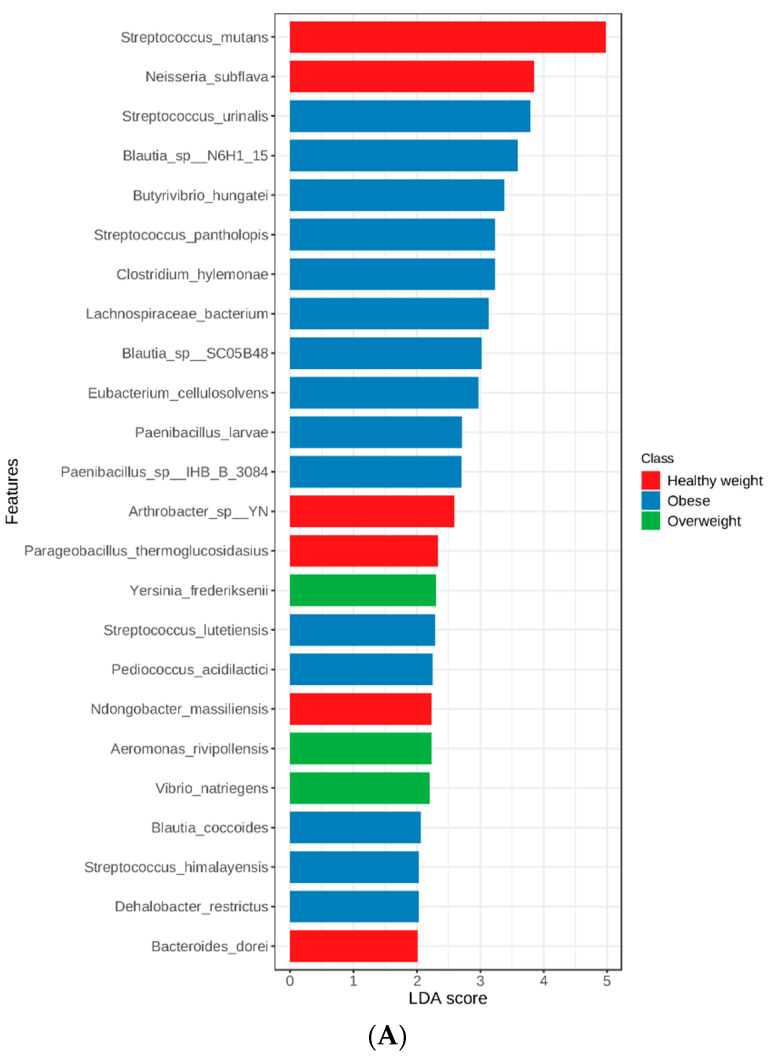

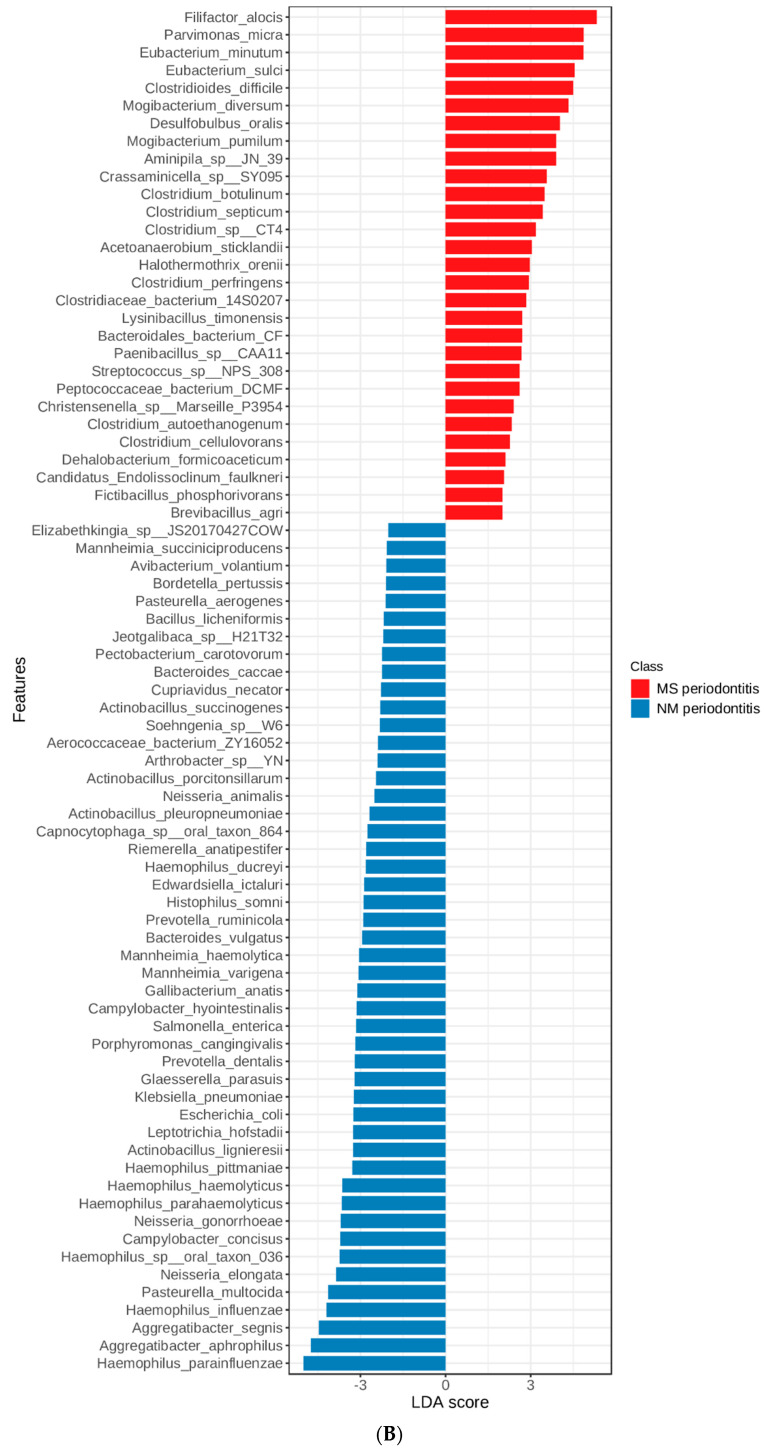

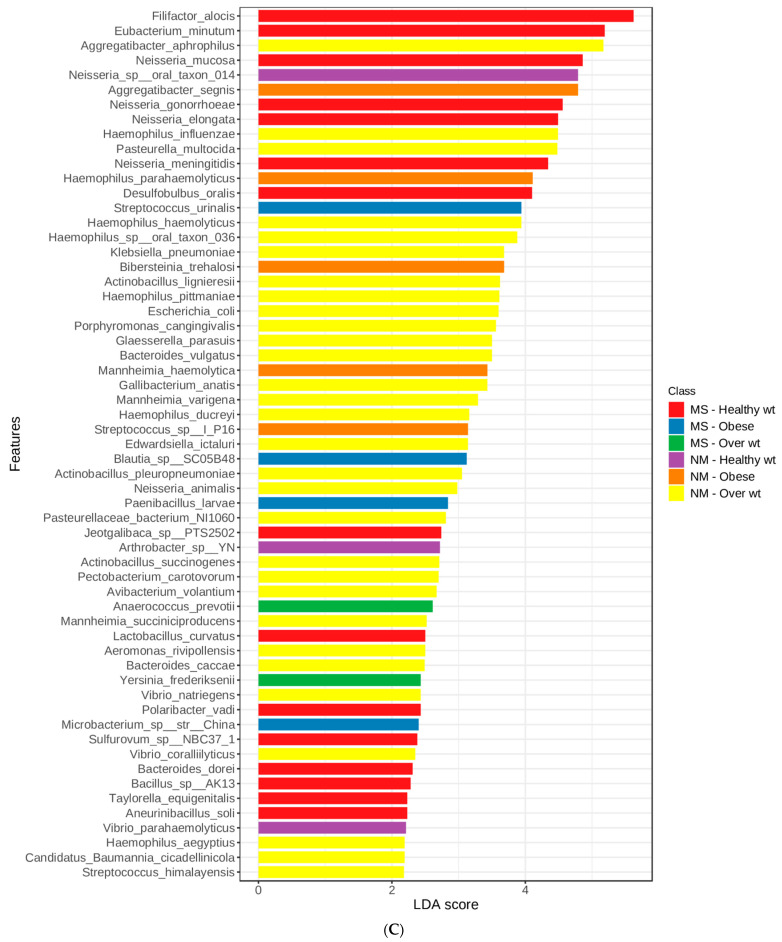
Species detected by linear discriminant analysis (LDA) effect size (LEfSe) in subjects grouped based on their BMI (**A**), periodontal health status (**B**), and both factors (**C**). The species shown are those that were significantly different among the groups ranked based on LDA score. Horizontal bars represent the effect size for each taxon.

Figure 5 shows the results of linear discriminate analysis (LDA) with scores for each species in subjects grouped based on their BMI (A) and also those with NM and MS periodontitis (B). As seen at the genus level, periodontal health status had a major impact on the species in the subgingival microbiome, with more differentially abundant species identified in subjects with NM than in those with MS periodontitis.

The top species detected were *Filifactor alocis, Eubacterium minutum*, and other species of *Neisseria* in the MS periodontitis group with healthy weight. The top species were *Aggregatibacter aphrophilus*, *Haemophilus influenzae*, and *Pasteurella multocida* in the NM periodontitis group with overweight, with multiple other potentially pathogenic species, such as *E. coli* and *Klebsiella pneumoniae. Aggregatibacter segnis* was the top species in the NM periodontitis group with obesity.

As for species in subjects grouped based on BMI, overweight and obese participants had more differentially abundant species than subjects with healthy weight. When both periodontal health status and BMI were considered (Figure 5C), it is obvious that overweight subjects with NM periodontitis had the highest number of unique species (*n* = 30) compared to the other groups, while 2–15 species were unique in the other groups (*p* < 0.05).

**Figure 6:**
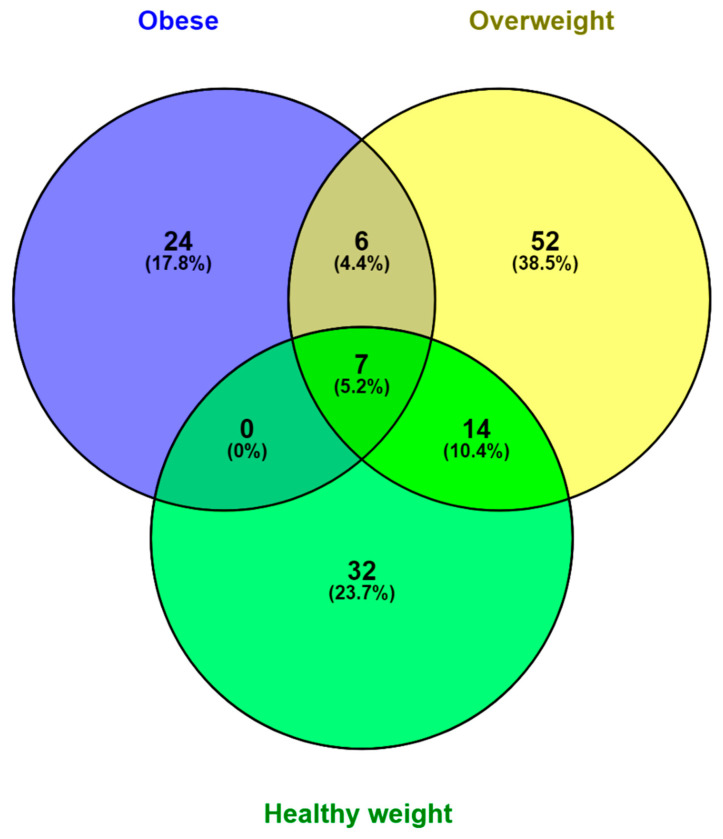
Venn diagram of shared and unique species detected in subgingival samples of subjects grouped based on BMI compared by their periodontal status. The numbers shown represent significantly different species between subjects with no-mild compared to moderate-severe periodontitis.

Subsequently, biomarker species detected in subjects with MS and NM periodontitis were compared based on BMI groups. The number of significantly different species are shown in the Venn diagram (Figure 6).

A total of 7 common species were shared among obese, overweight, and healthy weight subjects, namely *Haemophilus haemolyticus*, *Actinobacillus succinogenes*, *Haemophilus ducreyi*, *Mannheimia varigena*, *Haemophilus aegyptius*, *Actinobacillus lignieresii*, and *Glaesserella parasuis*. These species represented shared species that were significantly different between NM and MS periodontitis (*p* < 0.05). Other species were shared between the overweight group and either the obese or healthy weight groups, including 14 common species shared between overweight and healthy weight subjects, namely *Haemophilus influenzae*, *Haemophilus* sp. oral taxon 036, *Eubacterium minutum*, *Desulfobulbus oralis*, *Mannheimia haemolytica*, *Filifactor alocis*, *Haemophilus pittmaniae*, *Bibersteinia trehalose*, *Pasteurella multocida*, *Clostridium argentinense*, *Pasteurellaceae bacterium* NI1060, *Clostridioides difficile*, *Actinobacillus porcitonsillarum*, and *Peptococcaceae bacterium DCMF*. On the other hand, 6 species were shared between overweight and obese subjects, including *Mannheimia succiniciproducens*, *Porphyromonas cangingivalis*, *Pasteurella aerogenes*, *Tannerella* sp. oral taxon HOT-286, *Bacteroides vulgatus*, and *Chitinolyticbacter meiyuanensis*. As shown in Figure 6, the overweight group had the highest number of species with significant differences between NM and MS periodontitis (*p* < 0.05), reflecting higher diversity of the oral microbiome.

### 3.4. Heatmaps for Determination of Microbial Signatures

**Figure 7:**
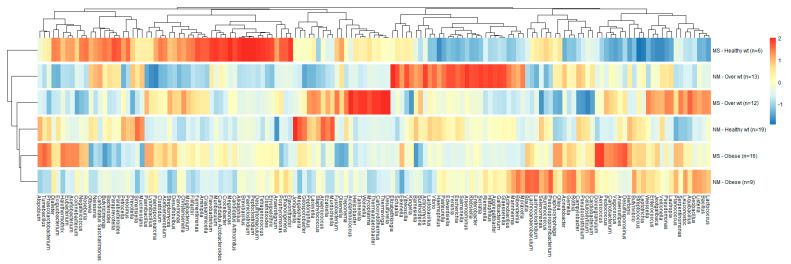
Heatmap showing the distribution of different genera (relative abundance  >  0.01%) grouped by BMI and periodontal condition. Relative abundances of genera were ranked based on group counts (scaled across columns). The number of participants in each group is also shown. Red indicates high abundance in a particular group and blue indicates low abundance. Dendrograms show clustering based on the relative abundance of different genera (left dendrogram: group clustering; top dendrogram: clustering of genera based on abundances in different groups).

Our results further confirmed that the microbiota in subjects with different BMIs and periodontal health status have distinct taxonomical signatures. A heatmap of the genera detected in at least 0.01% of the samples grouped based on both BMI and periodontal conditions was constructed, as shown in Figure 7.

As shown in Figure 7, some genera were enriched (dark orange) and others were depleted (light blue). The enrichment and depletion of genera was very obvious in the group of healthy weight subjects with MS periodontitis, which was different from the group of healthy weight subjects with NM periodontitis. Overweight subjects were unique, with very clear variations between those with MS compared to those with NM periodontitis. As for obese subjects, those with MS and NM periodontitis were similar (based on the dendrogram on the left of Figure 7), with obvious depletion of multiple genera compared to overweight and healthy weight subjects with similar periodontal conditions.

**Figure 8:**
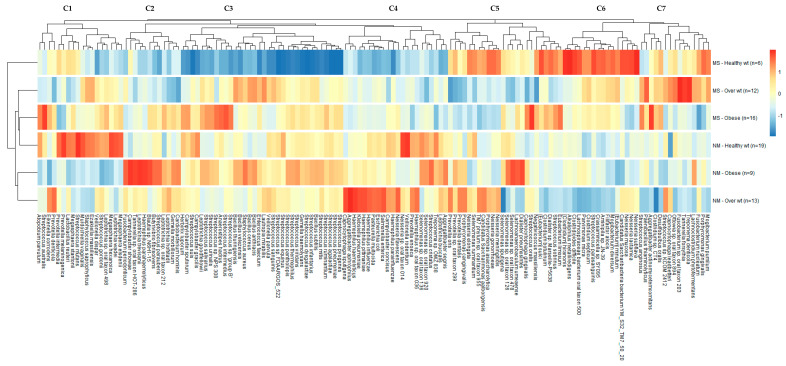
Heatmap showing the distribution of different species (relative abundance  >  0.1%) grouped by BMI and periodontal condition. Relative abundances of species were ranked based on group counts (scaled across columns). The number of participants in each group is also shown. Red indicates high abundance in a particular group and blue indicates low abundance. Dendrograms show clustering based on the relative abundance of different species (left dendrogram: group clustering; top dendrogram: clustering of species based on abundances in different groups).

Figure 8 shows a heatmap of species detected at an average abundance of >0.1% in the samples grouped based on BMI and periodontal health status. The heatmap clearly demonstrates the variations in species abundance in different groups with distinct taxonomical signatures and clustering patterns showing 7 unique clusters (C1-C7). As for healthy weight subjects, it was obvious that having MS periodontitis had a major impact with depletion of multiple species and enrichment of other species, forming a unique cluster compared to the other groups. As for overweight subjects, those with NM periodontitis exhibited some similarity to obese subjects with the same periodontal conditions, as both groups clustered together (dendrogram on the left). One the other hand, obese subjects with MS periodontitis had a unique signature with some similarity to overweight subjects with MS periodontitis. Overall, each group had a distinct microbiota signature compared to the other groups with a clear effect of both obesity and altered periodontal conditions.

Looking at the enriched species (Figure 8), the majority of periopathogenic bacteria (*P. gingivalis*, *T. forsythia*, *T. denticola*, *Fusobacterium nucleatum*, and *Aggregatibacter actinomycetemcomitans*) clustered together (cluster C7) and were enriched in subjects with MS periodontitis. As for the obese group, only *Aggregatibacter actinomycetemcomitans* showed the highest enrichment, being more enriched than in all the other groups, while *T. forsythia* and *T. denticola* showed the highest enrichment in the overweight MS periodontitis group. *P. gingivalis* and *Fusobacterium nucleatum* showed the highest enrichment in the healthy weight MS periodontitis group. It is noteworthy that the latter group had enrichment of many other species in clusters C5 and C6, including important oral pathogens such as *Filifactor alocis*, *Parvimonas micra*, and *Streptococcus sobrinus*. There was a clear depletion of multiple species of the normal flora belonging to the genus *Streptococcus* (cluster C3) in the healthy weight MS periodontitis group.

Other periopathogenic bacteria clustered together (cluster C1 in Figure 8), including *Eikenella corrodens*, *Prevotella intermedia*, and *Prevotella denticola*, were enriched in the obese MS periodontitis group and overweight NM periodontitis group. The same cluster included *Veillonella dispar*, *Streptococcus mutans*, and *Streptococcus gordonii* that were enriched in the healthy weight NM periodontitis group. As for the obese group with NM periodontitis, some species of the genus *Fusobacterium* and *Tannerella*, among others, were enriched (cluster C2). In cluster C4, some pathogenic bacteria, such as *E. coli*, *Salmonella enterica*, *Klebsiella pneumoniae*, and *Haemophilus influenzae*, were enriched in the overweight NM periodontitis group, with less enrichment in the other NM groups of obese and healthy weight. As for bacteria possibly related to obesity, species of the genus *Selenomonas* were enriched in the obese group with NM periodontitis in clusters C4 and C5.

### 3.5. Diversity

**Figure 9:**
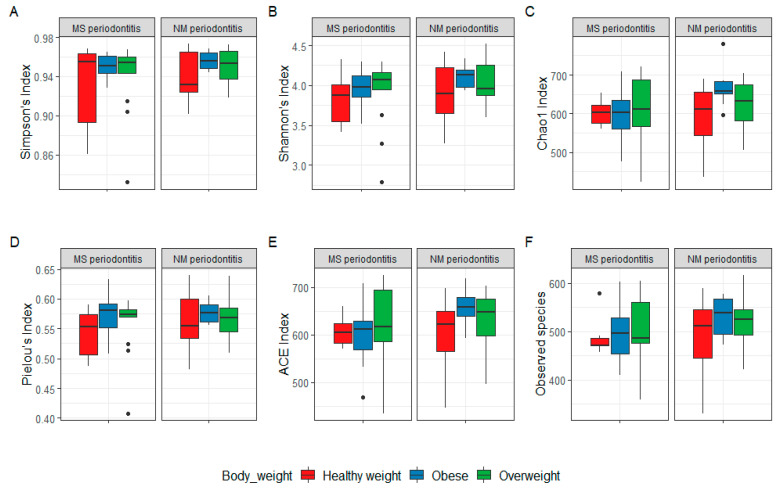
Alpha diversity in 75 subgingival plaque samples grouped based on periodontal health status and BMI. Shannon’s index (**A**), Simpson’s index (**B**), Chao1 index (**C**), Pielou’s index (**D**), ACE (**E**), and observed species (**F**). Box plots show Q1-median-Q3 with data range. Black dots are outlier values. MS: moderate-severe periodontitis, NM: no-mild periodontitis.

As for alpha diversity, there was a non-significant difference (*p* > 0.05) in all diversity measures when subjects were compared based on their periodontal health status or BMI. However, when subjects were sub-grouped based on BMI and compared, there was a significant difference in Chao1 and ACE diversity indices in the obese group (*p* < 0.05), as subjects with MS periodontitis had lower indices than those with NM periodontitis. All of the other indices were also lower in obese subjects with MS periodontitis, but the difference was not statistically significant (*p* > 0.05), reflecting that obese subjects with MS periodontitis had less diverse microbiomes than those with NM periodontitis (Figure 9).

For subjects with NM periodontitis, the Chao1 index was significantly lower in healthy weight subjects than in obese subjects (*p* < 0.05).

The correlation analysis did show any significant results, except for Chao1 index with body weight and Pielou’s index with both hip and waist circumferences (positive correlation, *p* < 0.05).

**Figure 10:**
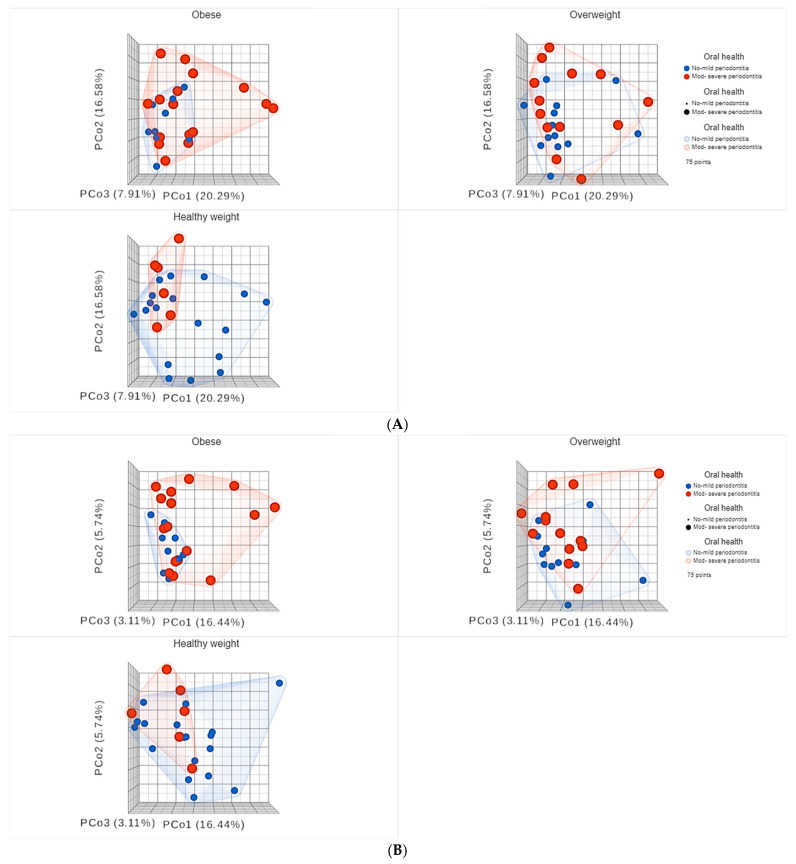
Principal coordinates (PCo) analysis plots of beta diversity indices: (**A**) Bray Curtis and (**B**) Jaccard. Participants were grouped based BMI, then sub-grouped based on periodontal heath status.

**Figure 11:**
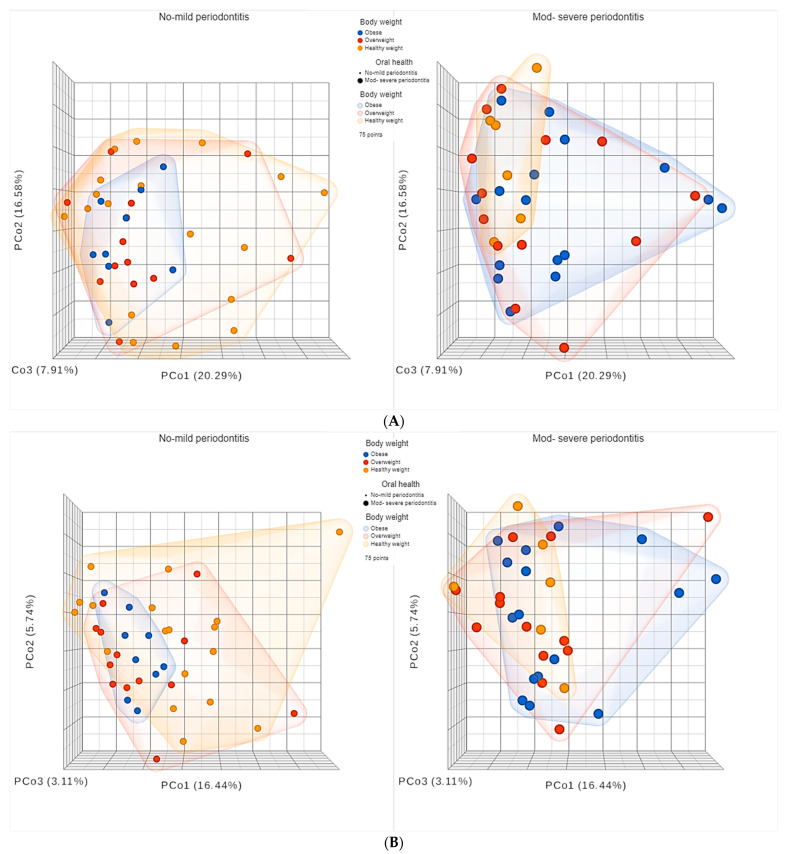
Principal coordinates (PCo) analysis plots of beta diversity indices (**A**) Bray Curtis and (**B**) Jaccard. Participants were grouped based periodontal heath status, then sub-grouped based on BMI.

As for beta diversity, the principal coordinates analysis (PCoA) plots are shown in Figure 10 and Figure 11.

Statistical comparison of beta diversity indices revealed significant differences between subjects with NM compared to those with MS periodontitis, as the PERMANOVA test for Bray–Curtis had an F-value of 2.2747, R-squared value of 0.030218 and *p*-value < 0.05. The PERMANOVA test for Jaccard index had an F-value of 1.7643, R-squared value of 0.023598, and *p*-value < 0.05.

As shown in Figure 10, for subjects sub-grouped based on BMI, the difference between subjects having NM periodontitis compared to those with MS periodontitis was very clear in both obese and healthy weight groups. However, in the obese group, higher diversity was observed among subjects with MS periodontitis, in contrast to the healthy weight group where higher diversity was observed among those with NM periodontitis, which represented the majority of subjects in the healthy weight group.

As shown in Figure 11, for subjects sub-grouped based on periodontal health, it was very obvious that obese subjects with NM periodontitis had less diverse microbiomes, with smaller clusters, than overweight and healthy weight subjects with NM periodontitis. As for subjects with MS periodontitis, healthy weight subjects had less diverse microbiomes, with smaller clusters, than obese and overweight subjects, who had highly diverse microbiomes. Statistical comparison of beta diversity indices revealed significant differences between healthy weight subjects and overweight and obese subjects sub-grouped based on having NM and MS periodontitis. The PERMANOVA test for Bray–Curtis had an F-value of 1.6139, R-squared value of 0.06384 and *p*-value < 0.05. The PERMANOVA test for Jaccard index had an F-value of 1.45, R-squared value of 0.057729, and *p*-value < 0.05. These results indicated that beta diversity was influenced by both BMI and periodontal health, causing significant variations in the subgingival microbiome.

## 4. Discussion

Obesity is recognized as a serious public health problem. According to the World Health Organization, more than 1.9 billion people are considered overweight, of which >650 million are obese [32]. Obesity and overweight are prevalent globally, including in the Middle East [1] and in the United Arab Emirates [33]. However, there is paucity of data on the complications of obesity and overweight in the Middle Eastern population, especially those related to oral health.

Several studies support a bi-directional relationship between obesity and periodontitis. The link was thought to be mediated by microbiome alterations. A significant association was found between obesity and poor periodontal health in this study, as participants with MS periodontitis had significantly higher BMI and WHR, with significant positive correlations between body weight, BMI, and PD as an indicator of periodontal health. These findings suggest that higher weight is associated with more severe periodontal disease, which supports the results of previous studies showing a correlation between periodontal disease and obesity [34,35,36]. In a Japanese population, the adjusted relative risk of periodontitis was 3.4 among those who were overweight and 8.6 among those who were obese, and the risk increased by 30% for every 5% increase in body fat [37]. Gorman et al. found that the risk for developing periodontitis was > 40% higher for those who were obese compared to their leaner counterparts, as assessed by both BMI and WHR [34]. Another prospective study found a significant association between periodontal disease and obesity even among non-diabetic individuals for all measures of adiposity, including BMI, waist circumference (WC), and WHR. Elevated hazard ratios for developing periodontitis were observed among those who were obese or had high WC or WHR [35]. In a meta-analysis of 57 observational studies, the prevalence odds of obesity was 33% higher for those with periodontitis across different populations from all around the world [36]. Al-Zahrani et al. reported an association between measures of obesity and periodontal disease among younger adults using data from the National Health and Nutrition Examination Survey III [38]. One major limitation of the previous studies was the lack of data on microbiome composition in obese and overweight populations, which was investigated in our study.

Dysbiosis refers to an imbalance of the microbiota, with a decrease in bacterial diversity and/or an increase of the relative abundance of certain pathogens. The dysbiotic gut microbiota in obesity was extensively discussed in a myriad of literature; however, limited data is available on the oral microbiome, although it is second to the gut microbiome in size. One of the measures of dysbiosis is the *Firmicutes/Bacteroidetes* (F/B) ratio, which has been associated with maintaining homeostasis and its change can lead to various pathologies. It has been reported that dysbiotic changes might not necessarily lead to a change in the F/B ratio [39]. Yang et al. did not find any significant difference in the levels of *Firmicutes* between obese and non-obese patients in mouth-rinse samples; however, they found that the family *Carnobacteriaceae*, genera *Gemella* and *Granulicatella*, and two species, *Granulicatella adiacens* and *Streptococcus oligofermentans*, were more abundant among obese participants [40].

Similarly, the *Firmicutes/ Bacteroidetes* ratio was not significantly different among the BMI or oral health groups in our study. However, *Firmicutes* was significantly higher in abundance in obese subjects than in overweight subjects, especially in subjects with NM periodontitis. This finding reflected that the increased abundance was related to high body weight rather than periodontal health, with subjects having early or very mild periodontitis in these cases. It is noteworthy that the phylum *Firmicutes* includes many known short-chain fatty acid-producing bacteria that provide an estimated 10% of the total dietary energy supply. A previous study reported that the relationship between *Firmicutes* and obesity was linked to a greater energy harvest. The increased abundance of species specializing in energy harvest due to alterations in the gut microbiota (increased *Firmicutes* and reduced *Bacteroidetes*) has been implicated in obesity in both animals and humans [41]. An increase in the abundance of *Firmicutes* was also seen in obese mice and mice fed a Western diet [42], suggesting that dietary factors in our study population might have impacted the oral microbiome, which is understudied in this part of the world.

Similar to our study, Tam et al. reported the influence of obesity on the subgingival microbiome of periodontitis patients, demonstrating significant discriminative features between obese and non-obese patients, such as *Proteobacteria, Chloroflexi*, and *Firmicutes*, which were overrepresented in subgingival plaques of obese patients, and the noted absence of representatives from the phylum *Bacteroidetes* [43]. In our study, *Proteobacteria* was significantly more abundant in subjects with NM periodontitis. *Proteobacteria* is a major phylum of Gram-negative bacteria, which includes a wide variety of pathogenic genera, such as *Escherichia*, *Klebsiella*, *Salmonella*, *Haemophilus*, and many others [44]. When we deeply investigated the altered genera and species, some pathogenic bacteria, such as *E. coli*, *Salmonella enterica*, *Klebsiella pneumoniae*, and *Haemophilus influenzae*, were found to be enriched in the overweight NM periodontitis group, with less enrichment in the other NM groups of obese and healthy weight subjects. This finding is alarming, as these subjects had mild or no periodontitis, which pinpoints that the high abundance of these pathogenic bacteria might indicate oral colonization in the overweight population, putting them at higher risk of systemic infections caused by these bacteria [45]. Previous studies demonstrated that a high-fat diet can increase lipopolysaccharide (LPS)-containing Gram-negative bacteria, which may explain the high abundance of *Proteobacteria* in overweight subjects [46]. LPS can induce metabolic endotoxemia and trigger downstream inflammation by interacting with CD14 cells and the co-receptor, Toll-like receptor 4 (TLR4), thus activating the nuclear factor κB (NF-κB) inflammatory pathway and leading to high transcription of several proinflammatory cytokines involved in the pathogenesis of chronic inflammation, which is a hallmark of obesity and other metabolic diseases [46].

When we performed linear discriminant analysis effect size (LEfSe) on the microbial abundance profiles at the genus and species levels, both periodontal health status and BMI were considered, and many biomarker bacterial genera and species with significant variations were found in obese, overweight, and healthy weight subjects, as well as biomarkers related to oral health. Regarding the enrichment/depletion of genera, overweight subjects showed clear variations between those with MS and NM periodontitis. On the other hand, obese participants demonstrated obvious depletion of multiple genera compared to overweight and healthy weight groups with similar periodontal conditions. The heatmaps of genus and species clearly showed that the microbiota of subjects with different BMIs and periodontal health status had distinct taxonomical signatures. Interestingly, more periopathogenic bacteria were enriched in subjects with MS periodontitis and healthy weight, namely *P. gingivalis*, *Fusobacterium nucleatum*, *Filifactor alocis*, *Parvimonas micra*, and *Streptococcus sobrinus*. Furthermore, there was a clear depletion of multiple species of the normal flora belonging to the genus *Streptococcus*. This reflects that more pathogenic bacteria are needed to initiate periodontal disease in healthy weight subjects, with the absence of underlying inflammation present when the body weight is high, which probably increases the risk of periodontal disease even with less pathogenic bacteria [13]. On the other hand, other types of periopathogenic bacteria were enriched in obese subjects (*Aggregatibacter actinomycetemcomitans*) and overweight subjects (*T. forsythia* and *T. denticola*) with MS periodontitis. There are studies reporting variations in the prevalence and abundance of microbiota affected by body weight and related to the periodontal condition. A previous study in the UAE also confirmed high levels of periopathogenic bacteria (*Fusobacterium* spp., *P. gingivalis*, and *T. forsythia*) found in significantly higher quantities in the saliva of obese patients [47]. Suresh et al. also reported that obese individuals with periodontitis harbored increased abundance of red complex bacteria [48]. Haffajee and Socransky in 2009 published the first study on subgingival biofilm composition and its relationship with obesity and periodontal disease. They found that only the abundance of *Tannerella forsythia* was significantly higher in obese but periodontally healthy participants [13]. Since then, only a few papers have investigated the subgingival microbiome of obese and non-obese patients with and without periodontal disease using various methods. Maciel et al. used checkerboard DNA-DNA hybridization to analyze subgingival biofilm samples in obese patients with and without periodontitis, reporting that proportions of *Aggregatibacter actinomycetemcomitans*, *Eubacterium nodatum*, *Fusobacterium nucleatum*, *S. vincentii*, *Parvimonas micra*, *Prevotella intermedia*, *Tannerella forsythia*, *Prevotella melaninogenica*, and *Treponema socranskii* species were increased in the diseased sites of obese patients compared to those with normal weight [49].

Silva-Boghossian et al. compared the composition of the subgingival microbiota between obese and non-obese women with or without periodontal disease, finding that *Porphyromonas gingivalis* and *Leptotrichia buccalis* were in higher counts in obese women than in non-obese women, while obese patients with periodontitis had larger amounts of *Capnocytophaga ochracea* than non-obese women with periodontitis. In addition, obese women with periodontitis showed significantly higher counts of *P. gingivalis* and *Tannerella forsythia* than non-obese women with healthy periodontium. Moreover, they also noticed that when the conditions obesity and periodontal disease were present at the same time, significant positive correlations were detected in levels of *Capnocytophaga ochracea*, *P. gingivalis*, *S. sanguinis*, and *T. forsythia* [50].

Previous studies reported that 98.4% of overweight women had a unique bacterial species, known as *Selenomonas noxia*, as a minor component of the salivary microbiota identified and enumerated by DNA probe analysis [14]. The latter study did not use sequencing; thus, it is possible that other species were missed. *Selenomonas noxia* was also detected in the subgingival biofilm of obese children [51]. In contrast, we could not detect this organism in our study, but other species from the genus *Selenomonas* were enriched in the obese group with NM periodontitis.

In a pilot study comparing healthy weight and obese subjects, obesity was associated with poor oral health, represented by an increase in numbers of missing teeth and periodontal support loss associated with dysbiotic oral microbiota [52]. In the latter study, alpha diversity was significantly reduced, in particular the Chao1 index. Similar findings were also reported in this study for Chao1 and ACE diversity indices in the obese group with MS periodontitis. This suggests a reduction in diversity due to the combined effect of obesity and severe periodontal disease. For subjects with NM periodontitis, the Chao1 index was also significantly lower in healthy weight subjects than in obese subjects. It was obvious that mild periodontal disease caused a shift in microbial diversity in healthy weight subjects due to microbiota depletion, which was also seen in the heatmaps of both genus and species, while obese and overweight subjects with NM periodontitis exhibited enrichment of many species with pathogenic potential. Nevertheless, there are reports suggesting that the diversity of the subgingival microbiome increases with the severity of periodontal disease [53]. We also tried to test the correlation between anthropometric measures of obesity and alpha diversity, but statistical analysis did show any significant results, except for a positive correlation between Chao1 index and body weight but not BMI, and Pielou’s index with both hip and waist circumferences but not WHR. This contradicts previous studies reporting that increased BMI was associated with a significant reduction in observed species, Chao1, and Shannon indices and a significant increase in the Simpson index, leading to the conclusion that BMI was negatively correlated with species richness and diversity in subgingival plaque [43]. Another study also reported an inverse relationship between BMI and the alpha diversity (Chao1) of the oral microbiota [54].

As for beta diversity, our results indicated that diversity is influenced by both BMI and periodontal health, causing significant variations in the subgingival microbiome. Interestingly, significant differences were found between subjects with NM compared to those with MS periodontitis, highlighting the peculiar effect of periodontal health. The effect was very obvious among obese subjects, as higher diversity was observed among subjects with MS periodontitis, in contrast to the healthy weight group, where higher diversity was observed among those with NM periodontitis, which represented the majority of subjects in the healthy weight group. On the contrary, obese subjects with NM periodontitis had less diverse microbiomes, with smaller clusters, than overweight and healthy weight subjects. As for subjects with MS periodontitis, healthy weight subjects had less diverse microbiomes, with smaller clusters, than obese and overweight subjects who had highly diverse microbiomes. This provides strong evidence that both obesity and overweight alter the diversity of the oral microbiome with a unique effect and microbial signatures.

It is noteworthy that a previous study confirmed the significance of the oral microbiome by comparing the salivary and fecal microbiota of the same subjects [55]. They found that the salivary microbiota provided a more distinct pattern for differentiation between obese and healthy weight individuals and was superior to the fecal microbiota. Although healthy and overweight groups were not distinct from each other, *Actinomyces* and *Haemophilus* were characteristic for the overweight group [55]. A recent study has proven that oral diseases, particularly periodontitis, can endanger the entire body by inducing gut microbiota dysbiosis and intestinal inflammation through the translocation of salivary microbes [56]. Considering that adults swallow ~10^12^ bacteria per day [57] and that saliva-derived microorganisms may colonize the gut, the important mechanism underlying changes in the gut microbiota of patients with periodontitis may be related to the entry of periodontal bacteria into the intestine via saliva through the oral-gut axis. Furthermore, the oral microbiota may contribute to systemic diseases via entry into the circulatory system through diseased periodontal tissues, also known as the oral-blood axis, or possibly through respiratory aspiration that can lead to pulmonary diseases [58]. Of course, microbial systemic penetration can be caused by mechanical injury such as dental procedures or during routine oral care activities, such as flossing and brushing, and even during mastication [59]. Examples of some pathogens reported in this study and implicated in systemic diseases are *A. actinomycetemcomitans* which can cause respiratory tract infections, and *F. nucleatum* and *P. gingivalis*, which can colonize the placenta inducing inflammation associated with fetal loss in human and animal studies [59]. Additionally, the microbes themselves might induce an intense inflammatory response and alter important cellular functions in the host. Examples of some pathogens reported in this study are *P. gingivalis* and *S. sanguis*, which can induce platelet aggregation and increase the risk of infarction. *P. gingivalis* can also modify low-density lipoproteins, thus enhancing foam cell formation and leading to atherosclerotic disease [59]. Some bacteria may have oncogenic potential, such as *F. nucleatum*, which is known as a promotor of colorectal cancer, and *Klebsiella* species, which can induce inflammation in the gut. Indeed, these bacteria clearly cause disruption of normal tissue homeostasis, which is a prerequisite for the initiation of many systemic diseases.

Crosstalk between the microbiota and different organs and systems is thought to be mediated by many pathways, such as cytokine communication via inflammatory response, trafficking of activated T cells, and bacterial metabolites. Periodontitis and its microbial components have an impact on host metabolism and immunity, for example, *P. gingivalis* was found to drive insulin resistance via an impaired adaptive immune response [60]. Thus, oral dysbiosis and colonization by pathogenic bacteria in association with obesity are serious issues that can lead to many adverse health complications.

Because oral dysbiosis was detected in obese and overweight populations, the use of probiotics may reduce the incidence of dysbiosis and improve oral health. This approach was described by other investigators who used probiotics to improve oral health and control multiple oral diseases [61,62], including periodontal disease [63,64]. The findings of these studies prove that dysbiosis is implicated in disease pathogenesis; therefore, improvement of oral health was achieved when dysbiosis was reversed using probiotics.

Ethnic variability as well as geographical location affect gut and oral microbiome composition [65], which can be also altered by obesity and overweight [66,67]. However, a previous study demonstrated that ethnicity-associated differences in the gut microbiota were stronger in lean subjects, while a difference in the gut microbiota was not shown in obese subjects from various ethnic groups. These findings suggested that established obesity or its associated dietary patterns can be the major determinant of long-lasting microbial composition [67]. Thus, the data reported in this study are a valuable addition to the existing knowledge of the oral microbiome and its relationship with obesity and overweight in the Middle Eastern population. Indeed, other factors influencing the oral microbiome should be explored in future studies.

Limitations of the study included difficulty in recruiting obese and overweight subjects without reported health issues; thus, sample collection took 2 years. Additionally, patient recruitment stopped due to the COVID-19 pandemic, despite the fact that collection of a bigger sample size was intended, which explains the low sample size per group. Finally, samples with low quality and/or quantity of DNA were excluded from the analyses to ensure the validity and accuracy of the next-generation sequencing data.

## 5. Conclusions

Obesity is a multidimensional problem, predisposing individuals to multiple complications, including oral diseases, as proven in this study. There is an intimate and significant correlation between measures of adiposity and predictors of periodontal disease, suggesting that higher body weight increases the risk for more severe periodontal disease. This is alarming since these diseases can cause local complications such as tooth loss, as well as systemic complications in other body sites related to the overgrowth of harmful bacteria in the oral cavity, which can be translocated to distant body sites. Furthermore, oral dysbiosis can lead to gut dysbiosis, which also predisposes individuals to serious health issues and numerous diseases. The identification of key pathogens altered in obesity and periodontal disease can help in the discovery of potential bacterial biomarkers that can be targeted during therapy or used to monitor therapeutic response. Next-generation sequencing of the microbial 16S rRNA gene allows the identification of multiple species and can provide unique bacterial signatures that can be used for diagnostic purposes [68]. Advanced genomic technologies and bioinformatics tools have provided a powerful means of understanding the contribution of the microbiome to human health. With the use of the portable sequencer (MinION), as in this study, it is possible to incorporate microbiota screening as part of assessments of oral and systemic health. Indeed, nutritional interventions, lifestyle modifications, and appropriate oral healthcare are recommended to mitigate the long- and short-term complications of oral dysbiosis. Modulation of the oral microbiome might be a promising future strategy to restore healthy balance in the oral cavity, which can also help in the management of obesity and prevention of its complications. Furthermore, proactive measures are required to increase awareness of the significance of oral health in the community, the importance of proper nutrition, and the need for a balanced diet for a healthy life, which can ultimately prevent obesity and its health-related issues.

## Figures and Tables

**Table 1:** Demographic and clinical characteristics of participants.

Characteristics		
**Continuous variables**	**Mean**	**Range**
Age (years)	31.1 ± 10.4	18–55
Body weight (kg)	84.4 ± 22.9	53–154.1
Hight (cm)	171.5 ± 9.3	153–190
BMI	28.4 ± 6.7	18.9–56.1
Waist circumference (cm)	45.3 ± 16.2	20.4–108
Hip circumference (cm)	49.6 ± 19.6	24.4–114
WHR	0.9 ± 0.09	0.7–1.2
**Categorical variables**	**N**	**%**
Gender		
- Female	16	21.3
- Male	59	78.7
Periodontal health		
- No-mild periodontitis	40	53.3
- Moderate-severe periodontitis	35	46.7

**Table 2:** Differences between participants with no-mild periodontitis versus moderate-severe periodontitis in parameters related to obesity and periodontal health.

Criteria	*Moderate-Severe Periodontitis*		*No-Mild Periodontitis*		*p* Value
	Mean ± SD	Range	Mean ± SD	Range	
**Anthropometric parameters**					
Waist circumference	44.10 ± 12.66	30.00–108.00	46.23 ± 18.83	20.40–102.00	0.414
Hip circumference	45.45 ± 12.37	36.60–110.00	53.04 ± 23.76	24.40–114.00	0.908
WHR *	0.97 ± 0.07	0.80–1.20	0.90 ± 0.09	0.7–1.09	0.007
Body weight	88.32 ± 21.45	60.00–154.10	81.06 ± 23.80	53.00–151.00	0.086
BMI *	30.08 ± 5.95	18.90–44.60	26.97 ± 7.05	19.15–56.10	0.008
**Periodontal health parameters #**					
PD *	2.96± 0.64	2.04–5.54	2.29 ± 0.57	1.10–3.96	<0.001
CAL *	1.76 ± 1.40	0.43–7.29	0.7 ± 0.42	0.00–2.77	<0.001
DMFT	7.47 ± 5.48	0–22	5.23 ± 4.8	0–17	0.067

* Significant difference. # For other periodontal health parameters (VPI and BOP): the difference was significant between NM and MS periodontitis, with higher scores (>25%) for the latter group (*p* < 0.01).

## Data Availability

Sequencing data have been deposited in Sequence Read Archive (SUB12513213).
